# COVID‐19 lockdowns affected birthing outcomes in a regional New South Wales Health District

**DOI:** 10.1111/ajo.13812

**Published:** 2024-04-02

**Authors:** Pierre Hofstee, Bridie Mulholland, Megan Kelly, Warren Davis, Kate Curtis

**Affiliations:** ^1^ Graduate School of Medicine, Faculty of Science, Medicine and Health University of Wollongong Wollongong New South Wales Australia; ^2^ The Tweed Hospital Northern New South Wales Local Health District Tweed Heads New South Wales Australia; ^3^ Faculty of Health Sciences and Medicine Bond University Gold Coast Queensland Australia; ^4^ School of Medical, Indigenous and Health Sciences University of Wollongong Wollongong New South Wales Australia; ^5^ Susan Wakil School of Nursing and Midwifery, Faculty of Medicine and Health University of Sydney Sydney New South Wales Australia; ^6^ Emergency Services Illawarra Shoalhaven Local Health District Wollongong New South Wales Australia

**Keywords:** maternal, neonatal, obstetrics, pregnancy, SARS‐CoV‐2

## Abstract

**Introduction:**

The 400 000 residents of the Illawarra Shoalhaven Local Health District (ISLHD) experienced two distinct lockdowns aimed at mitigating the transmission of severe acute respiratory syndrome coronavirus 2 infection. Analysing effects of these lockdowns on maternal and neonatal outcomes presents a valuable opportunity to assess the impact of pandemic‐level restrictions on maternal and neonatal outcomes.

**Aim:**

Evaluate the impacts of restrictions from two lockdown periods on maternal, birthing, and neonatal outcomes within a regional local health district.

**Materials and Methods:**

The study included 22 166 women who gave birth within ISLHD between 2017 and 2022. Groups included for analysis: Control Group – mothers pregnant before the pandemic (conception before 3 April 2019); Exposure Group 1 – mothers pregnant during the first lockdown (conception date 22 January 2020 to 5 May 2020); and Exposure Group 2 – mothers pregnant during the second lockdown (conception date 30 April 2021 to 13 Sep 2021).

**Results:**

Odds of adverse birthing outcomes including non‐reassuring fetal status (odds ratio (OR) 1.34; 95% CI 1.14–1.56 and OR 1.20; 95% CI 1.03–1.40), and postpartum haemorrhage (OR 2.04; 95% CI 1.73–2.41 and OR 1.74; 95% CI 1.48–2.05) were substantially increased in Exposure Groups 1 and 2, respectively. Gestational diabetes, gestational hypertension, low birth weight and admission to neonatal intensive care rates improved.

**Conclusions:**

Pregnant women exposed to pandemic restrictions within ISLHD had decreased odds of adverse antenatal and neonatal outcomes, but increased odds of poor peripartum outcomes.

## INTRODUCTION

The coronavirus disease 2019 (COVID‐19) pandemic required unprecedented public health measures. These were enforced to mitigate transmission of the virus and profoundly disrupted life in every aspect of society. Although the impact of severe acute respiratory syndrome coronavirus 2 (SARS‐CoV‐2) infection on pregnant women is still not entirely clear, the restrictions implemented to curb the transmission of SARS‐CoV‐2 have been linked to specific outcomes. For example, there were significant reductions in pre‐term birth (PTB) rates reported in various locations, initially in Ireland^1^ and Denmark.^2^

It is well established that the COVID‐19 lockdowns altered access to healthcare, increased stress, altered population lifestyles and had an impact on mental health within the collective pregnant community.^3^ The contraction of the virus itself is linked to increased risk of pre‐eclampsia, PTB, stillbirth, gestational diabetes mellitus (GDM) and low birth weight (LBW).^4^ Although the pandemic began in 2019, the first clinical obstetric recommendations were not announced until May of 2020 in the USA.^5^ Less is known of the impact of the lockdowns, independent of COVID‐19 infection, on maternal and neonatal outcomes. In 2021, Rolnik et al.^6^ investigated the impact of COVID‐19 restrictions on pregnancy duration and outcomes within a major Australian city; however, it is not well established as to how the lockdowns affected regional or rural Australia, where access to healthcare is inherently more difficult.

In 2020 and 2021 the 400 000 residents of the Illawarra Shoalhaven Local Health District (ISLHD), located along the regional coastline of New South Wales from Helensburgh to North Durras, were exposed to two separate and disparate lockdowns to ameliorate the transmission of SARS‐CoV‐2 infection. These lockdowns provide a unique opportunity to assess the impact of pandemic‐level restrictions on maternal and neonatal outcomes. Our aim was to compare the effects of restrictions from two distinct lockdown occasions on maternal, birthing, and neonatal outcomes within a regional local health district in Australia.

## MATERIALS AND METHODS

### Study setting and population

A retrospective cohort study was conducted using data from an Australian Local Health District. This study was approved by the ISLHD Low and Negligible Risk Committee (ISLHD/LNR/2021–139) and conducted in accordance with the National Statement on Ethical Conduct in Human Research (2007) and the Strengthening the Reporting of Observational Studies in Epidemiology guidelines.^7^

Data from all women aged 18 years and over who were pregnant and gave birth in the Illawarra Shoalhaven public system between 1 January 2017 and 1 December 2020 were included. Mothers with a multiple pregnancy and neonates with a birth defect, defined by the presence of an International Classification of Diseases‐Tenth Revision‐Australian Modification (ICD‐10‐AM) code, were excluded. Three groups were included for analysis: Control Group – mothers who were pregnant before the COVID‐19 pandemic (estimated conception date before 3 April 2019); Exposure Group 1 – mothers who were exposed to at least four weeks of restriction measures during their first trimester in the first lockdown period affecting the Illawarra Shoalhaven region (estimated conception date 22 January 2020 to 5 May 2020); and Exposure Group 2 – mothers who were exposed to at least four weeks of restriction measures during their first trimester in the second lockdown period affecting the Illawarra Shoalhaven region (estimated conception date 30 April 2021 to 13 September 2021).

**Figure 1:**
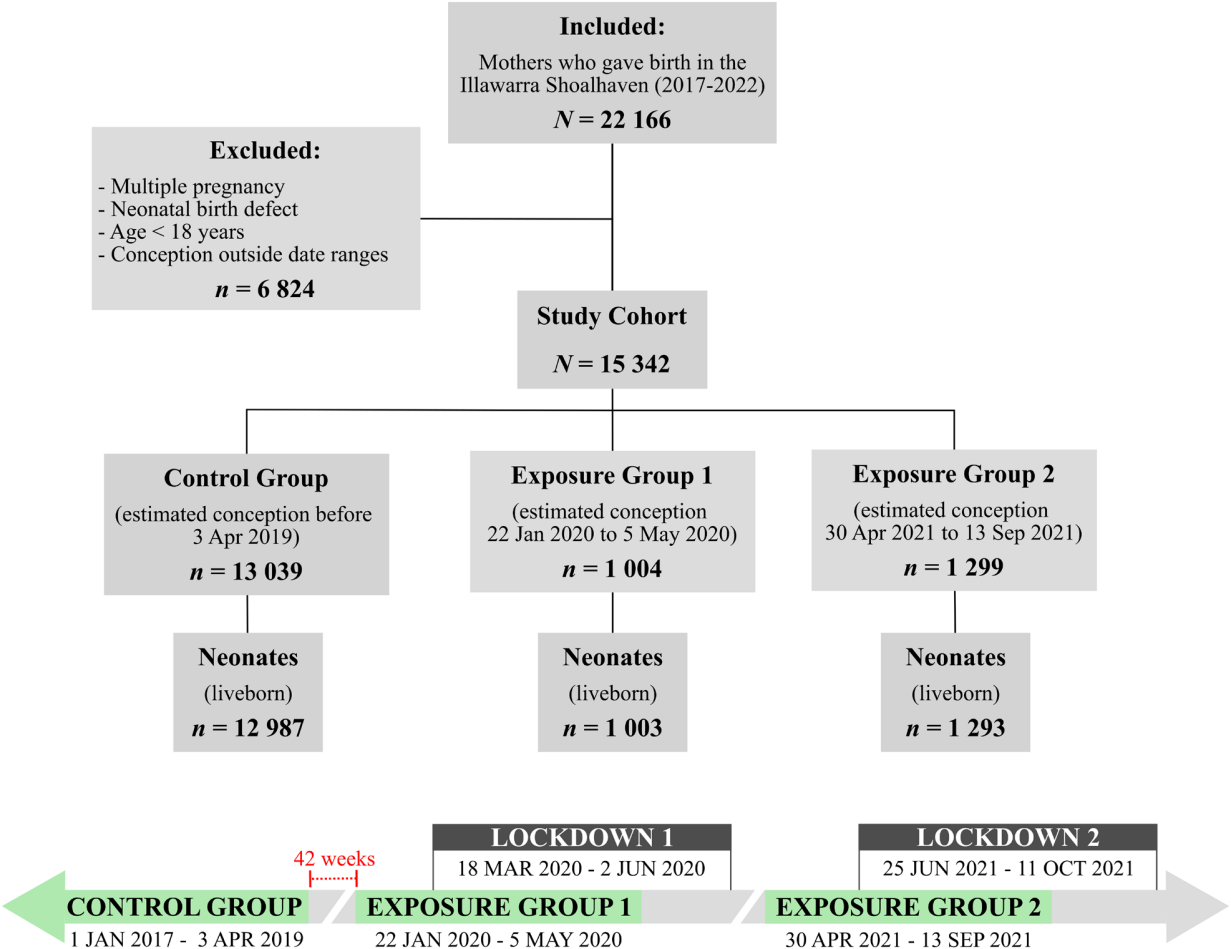
Flowchart of participants included in a study of women who were exposed to COVID‐19 restriction measures and unexposed controls.

The lockdown periods were defined per New South Wales Public Health Orders and are outlined in Fig. 1.^8^, ^9^ Restrictions were eased periodically; for this study, the lockdown periods were defined by the commencement and conclusion of stay‐at‐home orders specifically. An eight‐week and four‐week washout period were applied to the beginning and end of both lockdown periods, respectively. A 42‐week washout period from the start of Exposure Group 1 was applied to the control group.

### Outcome measures

ICD‐10‐AM codes from the mother's hospital admission for birth were used to create a baseline demographic and comorbidity electronic phenotype for each mother. Baseline demographics included: gender, maternal age (defined as age at admission for birth), Aboriginal or Torres Strait Islander status, gestational age, body mass index (BMI), parity and birth type. Caesarean section classification was defined using the Australian Classification of Health Intervention (fifth edition) code set representing procedures. Elective caesarean section refers to a caesarean section performed before the onset of labour; emergency caesarean section refers to a caesarean section performed after the onset of labour. All study outcomes were defined using the ICD‐10‐AM code set. The primary maternal outcomes were gestational hypertension, GDM and pre‐eclampsia; the primary birthing outcomes were elective and emergency caesarean, non‐reassuring fetal status and postpartum haemorrhage (PPH); the primary neonatal outcomes were PTB, LBW and admission to the neonatal intensive care unit (NICU). Non‐reassuring fetal status includes ICD‐10‐AM codes that refer to labour and delivery complicated by fetal stress, fetal heart rate anomaly, cord complications, cord prolapse, short cord, cord around neck, and meconium in amniotic fluid. Neonates weighing less than 2500 g were defined as LBW. Only live births were included for neonatal analysis.

### Statistical analyses

Relationships between categorical variables were compared using the χ^2^ test; continuous variables were compared using the Mann–Whitney *U*‐test or Kruskal–Wallis one‐way analysis of variance (ANOVA) as all continuous variables were either non‐parametric or did not have equal variances. Univariate and multivariable logistic regression were used to calculate the odds ratios (OR) and 95% confidence intervals (CI) for the primary maternal, birthing and neonatal outcomes. Multivariable models were adjusted for maternal age, BMI, parity, oligohydramnios, smoking, neonatal gender, GDM, pre‐eclampsia and gestational hypertension, except where any of the confounders were the dependent variable in the model. Additionally, the PPH model was also adjusted for birth type. Given the inferred effect of lockdowns on mental health and the unknown effects of COVID infection on pregnancy outcomes, multivariable models were also adjusted for maternal mental health condition (inclusive of depression and anxiety) and maternal COVID infection (inclusive of maternal history of COVID infection and maternal COVID infection at the time of birth). All analyses were conducted using Jamovi (version 2.3.16.0).

## RESULTS

In total, 22 166 women gave birth within the ISLHD between 2017 and 2022. After excluding multiple pregnancies, major neonatal birth defects and women below the age of 18, a population size of 21 561 women were included in the final analysis. A total of 1004 (Exposure Group 1; EG1) were exposed to restriction measures of the first lockdown; 1299 (Exposure Group 2; EG2) were similarly exposed to the second lockdown; and, 13 039 (control group) had an estimated conception date before 3 April 2019. After exclusion of stillbirths, there were 12 987 neonates in the control group, 1003 neonates in EG1, and 1293 neonates in EG2 (Fig. 1).

Maternal characteristics, maternal outcomes, birth characteristics, and birthing outcomes are outlined in Table 1. More women within both exposure groups were primiparous, with an increase of 13.74% in EG1 and 12.57% in EG2 and had a higher BMI (kg/m^2^). Rates of mental health conditions also differed. Depression increased in EG1 by 27.37% and in EG2 by 57.54%, whereas anxiety reduced by 28.00% in EG1 and by 50.40% in EG2. Regarding maternal outcomes, rates of prolonged pregnancy decreased by 31.97% in EG1 and 30.39% in EG2. Incidence of GDM also decreased by 23.07% in EG1 and by 16.08% in EG2, while gestational hypertension decreased by 25.12% in EG1 and 40.38% in EG2. Women in both exposure groups had significantly fewer vaginal births, with a reduction of 14.3% in EG1 and 22.67% in EG2. Elective caesareans increased by 9.94% in EG2, with emergency caesareans also increasing by 23.73% in the second lockdown. Non‐reassuring fetal status significantly increased by 42.73% in EG1 and 34.57% in EG2. Similarly, PPH increased by 89.49% in EG1 and 70.11% in EG2.

**Table 1:** Maternal characteristics, maternal outcomes, birth characteristics, and birth outcomes of women pregnant pre‐COVID (Control Group: estimated conception before 3 April 2019), during the first COVID lockdown (Exposure Group 1: estimated conception between 22 January 2020 and 5 May 2020) and during the second COVID lockdown (Exposure Group 2: estimated conception between 30 April 2021 and 13 September 2021)

	Control Group^a^	Exposure Group 1^b^	Exposure Group 2^c^	*P*‐value
	*n* = 13 039	*n* = 1004	*n* = 1299	
Maternal characteristics				
Maternal age, mean (SD)†	29.5 (5.34)	29.4 (5.00)	30.0 (5.07)^ab^	**0.004**
Indigenous, *n* (%)				
Indigenous	985 (7.55)	85 (8.47)	117 (9.00)	**0.250**
Non‐indigenous	12 049 (92.41)	918 (91.43)	1181 (90.92)	
Unknown	5 (0.04)	1 (0.10)	1 (0.08)	
Length of stay, days, mean (SD)†	2.88 (1.89)^bc^	2.63 (1.68)	2.62 (1.90)	**<0.001**
Parity, *n* (%)				
Primipara	3700 (28.38)	423 (42.13)	532 (40.95)	**<0.001**
Multipara	9339 (71.62)	581 (57.87)	767 (59.05)	
Body mass index, mean (SD)†	25.8 (6.14)^bc^	26.2 (5.99)	26.7 (6.27)	**<0.001**
Mental health condition, *n* (%)				
Depression	1019 (7.82)	100 (9.96)	160 (12.32)	**<0.001**
Anxiety	163 (1.25)	9 (0.90)	8 (0.62)	
Gestational age, mean (SD)†	39.0 (1.77)^c^	39.0 (1.46)	38.8 (1.80)	**<0.001**
Smoking during pregnancy, *n* (%)	889 (6.82)	66 (6.57)	94 (7.24)	0.802
Maternal outcomes				
Prolonged pregnancy, *n* (%)	1240 (9.51)	65 (6.47)	86 (6.62)	**<0.001**
Antepartum haemorrhage, *n* (%)	169 (1.30)	17 (1.69)	24 (1.85)	0.174
Gestational diabetes, *n* (%)	2465 (18.90)	146 (14.54)	206 (15.86)	**<0.001**
Gestational hypertension, *n* (%)	556 (4.26)	32 (3.19)	33 (2.54)	**0.007**
Pre‐eclampsia, *n* (%)	245 (1.88)	21 (2.09)	27 (2.08)	0.802
Polyhydramnios, *n* (%)	159 (1.22)	17 (1.69)	19 (1.46)	0.353
Oligohydramnios, *n* (%)	341 (2.62)	19 (1.89)	33 (2.54)	0.376
Chorioamnioitis, *n* (%)	22 (0.17)	4 (0.40)	4 (0.31)	0.179
Birth characteristics				
Birth type, *n* (%)				
Vaginal	7652 (58.69)	554 (55.18)	685 (52.73)	**<0.001**
Instrumental	1310 (10.05)	139 (13.84)	122 (9.39)	
Elective caesarean	1908 (14.63)	139 (13.84)	210 (16.16)	
Emergency caesarean	2176 (16.68)	169 (16.83)	275 (21.17)	
Prolonged labour, *n* (%)				
First stage	95 (0.73)	5 (0.50)	7 (0.54)	**0.005**
Second stage	580 (4.45)	29 (2.89)	42 (3.23)	
Birthing outcomes				
Non‐reassuring fetal status, *n* (%)	2276 (17.53)	251 (25.02)	305 (23.59)	**<0.001**
Postpartum haemorrhage, *n* (%)	1440 (11.04)	210 (20.92)	244 (18.78)	**<0.001**

† Significant differences from pairwise comparisons for continuous variables are indicated using a, b and c.

Significant *P*‐values are bolded.

SD, standard deviation.

Neonatal characteristics, outcomes and sex differences are represented in Table 2. There was no impact of either lockdowns on gross measures of neonatal characteristics, including neonatal sex, length, weight or head circumference. Regarding neonatal outcomes, LBW was significantly increased within EG2 by 18.2%, although admission to NICU was significantly reduced within both exposure groups (31.62 and 18.48% respectively). There was no change in rates of PTB, stillbirth, congenital conditions and small for gestational age between the groups.

**Table 2:** Characteristics, outcomes and sex differences of neonates born to women pregnant pre‐COVID (estimated conception before 3 April 2019), during the first COVID lockdown (estimated conception between 22 January 2020 and 5 May 2020) and during the second COVID lockdown estimated conception between 30 April 2021 and 13 September 2021)

	Control Group	Exposure Group 1	Exposure Group 2
	*n* = 12 987	*n* = 1003	*n* = 1293
Neonatal characteristics			
Sex, *n* (%)			
Male	6785 (52.04)	526 (52.39)	667 (51.35)
Female	6254 (47.97)	478 (47.61)	632 (48.65)
Length (cm), mean (SD)	50.3 (2.63)	50.5 (2.62)	50.4 (2.87)
Weight (g), mean (SD)	3408 (518)	3432 (495)	3379 (543)
Head circumference (cm), mean (SD)	34.4 (1.65)	34.4 (1.59)	34.4 (1.70)
Neonatal outcomes			
Pre‐term birth, *n* (%)	789 (6.05)	50 (4.98)	78 (6.00)
Stillbirth, *n* (%)^†^	52 (0.40)	1 (0.10)	6 (0.46)
Congenital condition, *n* (%)	63 (0.49)	2 (0.20)	2 (0.15)
Small for gestational age, *n* (%)	277 (2.13)	19 (1.89)	36 (2.78)
Low birthweight, *n* (%)	621 (4.78)	34 (3.40)	73 (5.65)**
Admission to NICU, *n* (%)	1799 (13.85)***	95 (9.47)	146 (11.29)

Sex differences	Male	Female	Male	Female	Male	Female
	*n* = 6785	*n* = 6254	*n* = 526	*n* = 478	*n* = 667	*n* = 632
Pre‐term birth, *n* (%)	408 (6.04)	316 (5.2)	29 (5.51)	21 (4.40)	39 (5.88)	39 (6.19)
Stillbirth, *n* (%)^†^	29 (0.43)	23 (0.37)	0 (0)	1 (0.21)	4 (0.60)	2 (0.32)
Length (cm), mean (SD)	50.7 (2.65)	49.9 (2.55)***	50.9 (2.59)	49.9 (2.58)***	50.9 (2.64)	49.9 (3.01)***
Weight (g), mean (SD)	3465.8 (520.91)	3345.17 (506.81)***	3492.47 (485.10)	3364.30 (498.13)***	3459.45 (532.80)	3295.29 (541.43)***
Head circumference (cm), mean (SD)	34.71 (1.63)	34.16 (1.63)***	34.74 (1.47)	34.12 (1.65)***	34.73 (1.53)	34.05 (1.80)***
Small for gestational age, *n* (%)	199 (2.95)	78 (1.25)***	14 (2.66)	5 (1.05)	29 (4.37)	7 (1.11)***
Low birthweight, *n* (%)	287 (4.25)	334 (5.36)**	14 (2.66)	20 (4.19)	29 (4.37)	44 (6.98)*
Admission to NICU, *n* (%)^‡^	1009 (14.93)	790 (12.68)***	60 (11.41)	35 (7.34)*	88 (13.27)	58 (9.21)*

^†^ Calculated among all neonates (pre‐COVID = 13 039; COVID lockdown 1 = 1004; COVID lockdown 2 = 1299).

^‡^ Sex difference between groups, admission to NICU was significantly different between groups in females but not males.

Significant values are bolded. Sex differences within groups are bolded. Level of significance is indicated using asterisks (*P < 0.05, **P < 0.01, ***P < 0.001).

NICU, neonatal intensive care unit; SD, standard deviation.

Table 3 represents the association between the exposure to COVID lockdowns during pregnancy on maternal, birthing and neonatal outcomes. When adjusted for confounding variables, the odds of GDM were reduced (OR 0.73; 95% CI 0.61–0.88) in EG1, with the odds of both gestational hypertension (OR 0.60; 95% CI 0.41–0.88) and GDM (OR 0.77; 95% CI 0.65–0.91) significantly reduced in EG2. The odds of primary birthing outcomes were also affected by lockdowns. Both non‐reassuring fetal status (OR 1.34; 95% CI 1.14–1.56 and OR 1.20; 95% CI 1.03–1.40 respectively) and PPH (OR 2.04; 95% CI 1.73–2.41 and OR 1.74; 95% CI 1.48–2.05 respectively) increased in both EG1 and EG2 with the rates of elective caesarean increasing in EG2 (OR 1.34; 95% CI 1.13–1.58), and no change in emergency caesareans. Irrespective of this, the odds of neonatal LBW (OR 0.69; 95% CI 048.‐0.99) and admission to NICU (OR 0.65; 95% CI 0.52–0.80) were significantly reduced in EG1, with admission to NICU odds also reduced in EG2 (OR 0.82; 95% CI 0.67–0.99).

**Table 3:** Association between exposure to COVID lockdowns during pregnancy and study outcomes

	Exposure Group 1		Exposure Group 2	
	Unadjusted OR (95% CI)	Adjusted OR (95% CI)	Unadjusted OR (95% CI)	Adjusted OR (95% CI)
Maternal outcomes				
Gestational hypertension	0.74 (0.51–1.06)	0.71 (0.50–1.02)	0.59 (0.41–0.84)**	0.60 (0.41–0.88)**
Gestational diabetes	0.73 (0.61–0.87)***	0.73 (0.61–0.88)***	0.81 (0.69–0.95)**	0.77 (0.65–0.91)**
Pre‐eclampsia	1.12 (0.71–1.75)	1.05 (0.67–1.65)	1.11 (0.75–1.66)	0.93 (0.59–1.47)
Birthing outcomes				
Elective caesarean	0.94 (0.78–1.13)	1.08 (0.89–1.31)	1.07 (0.89–1.31)	1.34 (1.13–1.58)***
Emergency caesarean	1.01 (0.85–1.20)	0.87 (0.73–1.04)	1.35 (1.17–1.55)***	1.14 (0.97–1.33)
Non‐reassuring fetal status	1.57 (1.35–1.83)***	1.34 (1.14–1.56)***	1.45 (1.27–1.66)***	1.20 (1.03–1.40)*
Postpartum haemorrhage	2.14 (1.82–2.52)***	2.04 (1.73–2.41)***	1.87 (1.61–2.17)***	1.74 (1.48–2.05)***
Neonatal outcomes				
Pre‐term birth	0.81 (0.61–1.09)	0.79 (0.59–1.06)	0.99 (0.78–1.26)	1.07 (0.84–1.37)
Low birthweight	0.70 (0.49–0.99)*	0.69 (0.48–0.99)*	1.19 (0.93–1.53)	1.27 (0.98–1.66)
Admission to NICU	0.65 (0.52–0.81)***	0.65 (0.52–0.80)***	0.79 (0.66–0.95)*	0.82 (0.67–0.99)*

Models were adjusted for maternal COVID infection, age, body mass index, parity, oligohydramnios, smoking, neonatal gender, maternal mental health condition, gestational diabetes mellitus, pre‐eclampsia and gestational hypertension, except where any of the confounders were the dependent variable in the model. Additionally, the postpartum haemorrhage model was also adjusted for birth type. Significant models are bolded. Level of significance is indicated using asterisks (*P < 0.05, **P < 0.01, ***P < 0.001).

CI, confidence interval; NICU, neonatal intensive care unit; OR, odds ratio.

## DISCUSSION

Examination of the outcomes of 21 561 pregnancies in the ISHLD, a regional district of NSW, demonstrates pregnant women subjected to COVID‐19 restrictions experienced unfavourable birthing outcomes while concomitantly experiencing improved maternal and neonatal outcomes. Rates of elective caesareans, non‐reassuring fetal status, and rates of PPH all increased. Birthing outcomes, including mode of delivery, have not previously been found to have been impacted by the pandemic or lockdowns. Number of caesarean deliveries has previously been shown to be unaffected (OR, 1.03; 95% CI, 0.99–1.07; 17 studies) in large cohort studies,^10^ with a significant reduction in length of hospital stay for both caesareans and vaginal births. In the present study, mean length of hospital stay was reduced for both lockdowns; however, rates of non‐reassuring fetal status, and rates of PPH were substantially increased, and are likely not reflective of the increased rates of caesareans, as both increased significantly in each exposure group.

Despite reporting a substantial increase in non‐reassuring fetal status in the present study, the reason for this increase is not clear. It is well established that maternal age, BMI, parity, oligohydramnios, smoking, neonatal gender, GDM, and gestational hypertension can increase the risk of non‐reassuring fetal status and a subsequent need for an emergency caesarean; however, these potential confounding variables were either improved or unchanged in EG1 and EG2 compared to the control group, and were controlled for in multivariable models, suggesting that the increased odds of non‐reassuring fetal status were not related to these commonly associated confounding variables. An important consideration is the potential effect the lockdowns had on depression and anxiety. During the ongoing COVID‐19 crisis it is commonly indicated that pregnant women experienced considerably increased levels of anxiety with additional increased rates of depression.^11^, ^12^ A recent meta‐analysis suggests anxiety in pregnant women during COVID‐19 was as high as 40%, with depression as significant as 27% in the antenatal period.^13^ In the current study, we observed an inverse relationship between the two with increased rates of depression and reduced levels of anxiety across the subsequent lockdowns. Given the purported effects of lockdowns on mental health, we adjusted our models for mental health, which had no effect on the ORs. However, it is noted that maternal stress status at birth was not able to be directly quantified in the present study, and that although women included in the study experienced a portion of their first trimester in lockdown, this does not mean they necessarily gave birth during a lockdown. Nonetheless, traumatic birth experiences can have lasting impacts on both mothers and babies, with established links to postpartum depression, reduced quality of parent–child interaction and poor maternal and child health.^14^

Unlike previous studies in Australia^6^ and internationally,^3^, ^10^ we did not find a change in the rates of PTB due to either lockdowns in NSW, albeit most evidence observing lower rates of PTB. Additional meta‐analyses have shown that GDM rates were not affected by the lockdown in both high income (OR, 1.02; 95% CI, 0.85–1.22; five studies) and low‐ and middle‐income countries (OR, 1.01; 95% CI, 0.60–1.71; one study).^10^ Here, we found a substantial improvement in the rates of GDM during both lockdowns. It is plausible that screening of maternal conditions decreased due to lockdowns, thus this rate decrease in GDM could be subsequent to underreporting in our cohort and changes in the diagnostic process.^15^ Rates of gestational hypertension were also significantly reduced, particularly within the second lockdown, with no change in the rates of pre‐eclampsia; however, there are currently no data indicating reduced screening and reporting of gestational hypertension, similar to GDM. The possibility of underreporting of antenatal conditions may also serve as a potential explanation for the increase in elective caesareans, although this is speculatory. Rolnik et al.^6^ in Australia, and several international studies,^16^, ^17^ also reported no changes in the incidence of pre‐eclampsia. This is contrary to a previous study in China, that reported increases in hypertensive disorders of pregnancy (OR, 5.59; 95% CI, 1.59–19.7).^18^ The variance in lockdown conditions from country to country, state to state and lockdown to lockdown, makes it difficult to make generalisations on the effect of pandemic restrictions on maternal, neonatal and birthing outcomes, and further highlights the importance of region‐specific analyses.

### Strengths and limitations

Given the substantial influence of the first trimester on pregnancy outcomes – and contrary to earlier studies investigating the effects of lockdowns on pregnancy – this study, to the authors’ knowledge, is the first to specifically focus on women who experienced a minimum of four weeks of their first trimester in a lockdown. We emphasise that these groups are defined by four weeks of first trimester exposure to lockdowns and our observations show that there may be early effects on embryogenesis and placenta development that can impact later pregnancy outcomes when no longer in lockdown. As the two subsequent lockdowns varied so greatly in onset and restriction levels, this study examined both lockdowns independently and, to the authors’ knowledge, is the first and only Australian study to have done so. It is important to note that this study has several limitations: the nature of retrospective cohort studies is one that is unable to delineate mechanisms or establish causative effect, so all associations show correlation only; routinely collected health data were used in the preparation of the dataset that was analysed for this study – accuracy of this type of data cannot be guaranteed; information on clinical decision‐making and medications data is lacking and would provide important contextual information, future studies should consider including data of this kind.

Our study confirms the presently held idea that pregnant women exposed to pandemic restrictions experienced lower rates of antenatal complications. Previous studies have focused on maternal and neonatal outcomes when considering the potential effect of COVID‐19 lockdowns on pregnancy, with little to no focus on birthing outcomes. We highlight a significant increase in peripartum complications in women who experienced at least four weeks of their first trimester during a lockdown, including increased odds of non‐reassuring fetal status and PPH. While reasons for this reported increase in peripartum complications is unclear in the current study, this finding is of note given the negative and long‐lasting ramifications a traumatic birth experience can have on mother and child. Further characterisation of the causative effect of lockdowns on antenatal and peripartum outcomes is paramount and may inform future obstetric care guidelines during unprecedented global events.

## AUTHOR CONTRIBUTIONS

All authors have approved the final version of the manuscript and agree to be accountable for all aspects of the work. All persons designated as authors qualify for authorship, and all those who qualify for authorship are listed.

## FUNDING

This research received no specific grant from any funding agency in the public, commercial, or not‐for‐profit sectors. The authors acknowledge the Illawarra Health Information Platform research partnership established between the Illawarra Shoalhaven Local Health District (ISLHD) and the University of Wollongong, with ISLHD providing funding, support and the data used in this study.

## ETHICS APPROVAL

Approval for this study was obtained from the ILSHD Low and Negligible Risk Committee (ISLHD/LNR/2021–139). This project used non‐identifiable data and, therefore, consent for participation was not required.

## Data Availability

The data that support the findings of this study are available from the ISLHD, but restrictions apply to the availability of these data, which were used under licence for the current study, and so are not publicly available.
